# Poly[[μ_3_-2-(benzotriazol-1-yl)acetato-κ^3^*O*:*O*′:*N*^3^]chlorido­(ethanol-κ*O*)cobalt(II)]

**DOI:** 10.1107/S2414314624006308

**Published:** 2024-07-05

**Authors:** Yun-Yun Zheng, De-Sen Su, Qing-Hua Yao, Min-Min Huang

**Affiliations:** a Fujian Key Laboratory of Agro-Products Quality and Safety Institute of Quality Standards Testing Technology for Agro-products, Fujian Academy of Agricultural Sciences 247 Wu-Si Rd Fuzhou People’s Republic of China; University of Aberdeen, United Kingdom

**Keywords:** 2-(benzotriazol-1-yl) acetic acid, cobalt, coordination polymers, crystal structure

## Abstract

In the title compound, [Co(C_8_H_6_N_3_O_2_)Cl(C_2_H_5_OH)]_*n*_, the Co^II^ atoms adopt octa­hedral *trans*-CoN_2_O_4_ and tetra­hedral CoCl_2_O_2_ coordination geometries (site symmetries  and *m*, respectively). The bridging μ_3_-*O*:*O*:*N* 2-(benzotriazol-1-yl)acetato ligands connect the octa­hedral cobalt nodes into (010) sheets and the CoCl_2_ fragments link the sheets into a tri-periodic network. The structure displays O—H⋯O hydrogen bonding and the ethanol mol­ecule is disordered over two orientations.

## Abstract 2

The reaction of 2-(benzotriazol-1-yl) acetic acid with CoCl_2_ at room temperature in ethanol generates the title three-dimensional cobalt-based coordination polymer.

## Graphical abstract

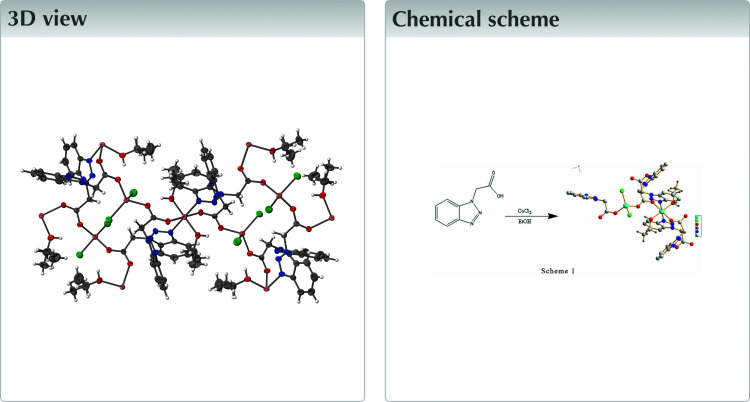

## Structure description

As a ligand with multiple coordination sites, benzotriazole is a good linker in the generation of metal–organic frameworks (MOFs) as it can bridge different metal cations to afford coordination polymers that exhibit structural diversity and facile accessibility of functionalized new magnetic materials (Bai *et al.*, 2008 ▸; Shao *et al.*, 2008 ▸; Müller-Buschbaum & Mokaddem, 2006 ▸). Functional groups such as carboxyl­ate, hy­droxy and pyridyl can be added to the benzotriazole core, increasing its coordination possibilities (Stoumpos *et al.*, 2008 ▸; Zhang *et al.*, 2007 ▸; Hu *et al.*, 2008 ▸; Hang & Ye, 2008 ▸). 1*H*-Benzotriazole-1-acetic acid (Hbtaa), a flexible ligand, containing a carboxyl­ate group (when deprotonated) and a triazole unit has been used to construct MOFs (Zheng *et al.*, 2010 ▸; Zeng, 2013 ▸). As part of our work in this area, we now report the synthesis and crystal structure of the title coordination polymer, [Co(C_8_H_6_N_3_O_2_)Cl(C_2_H_5_OH)]_*n*_, where C_8_H_6_N_3_O_2_^−^ (*L*^−^) is the 2-benzotriazol-1-yl)acetate anion.

**Figure 1:**
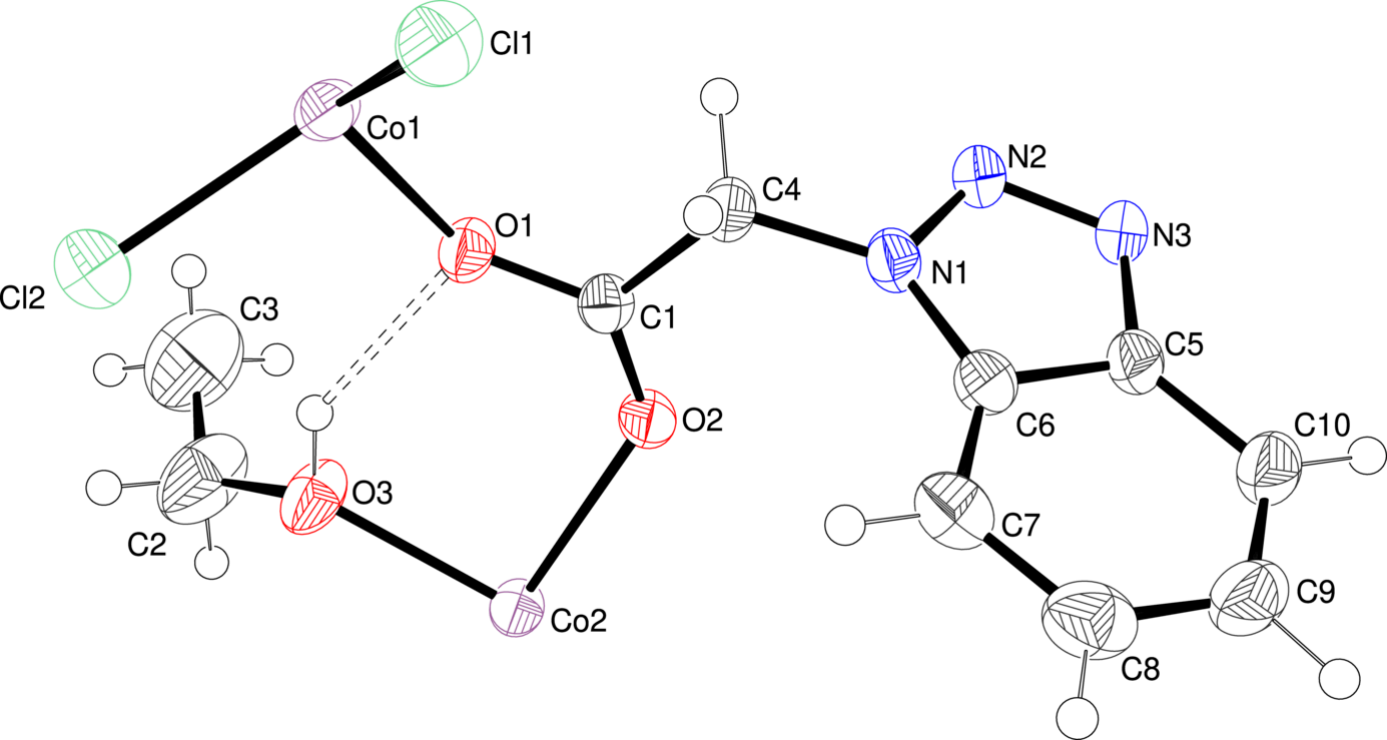
The asymmetric unit of the title compound showing 50% displacement ellipsoids. Only one orientation of the disordered ethanol mol­ecule is shown.

**Figure 2:**
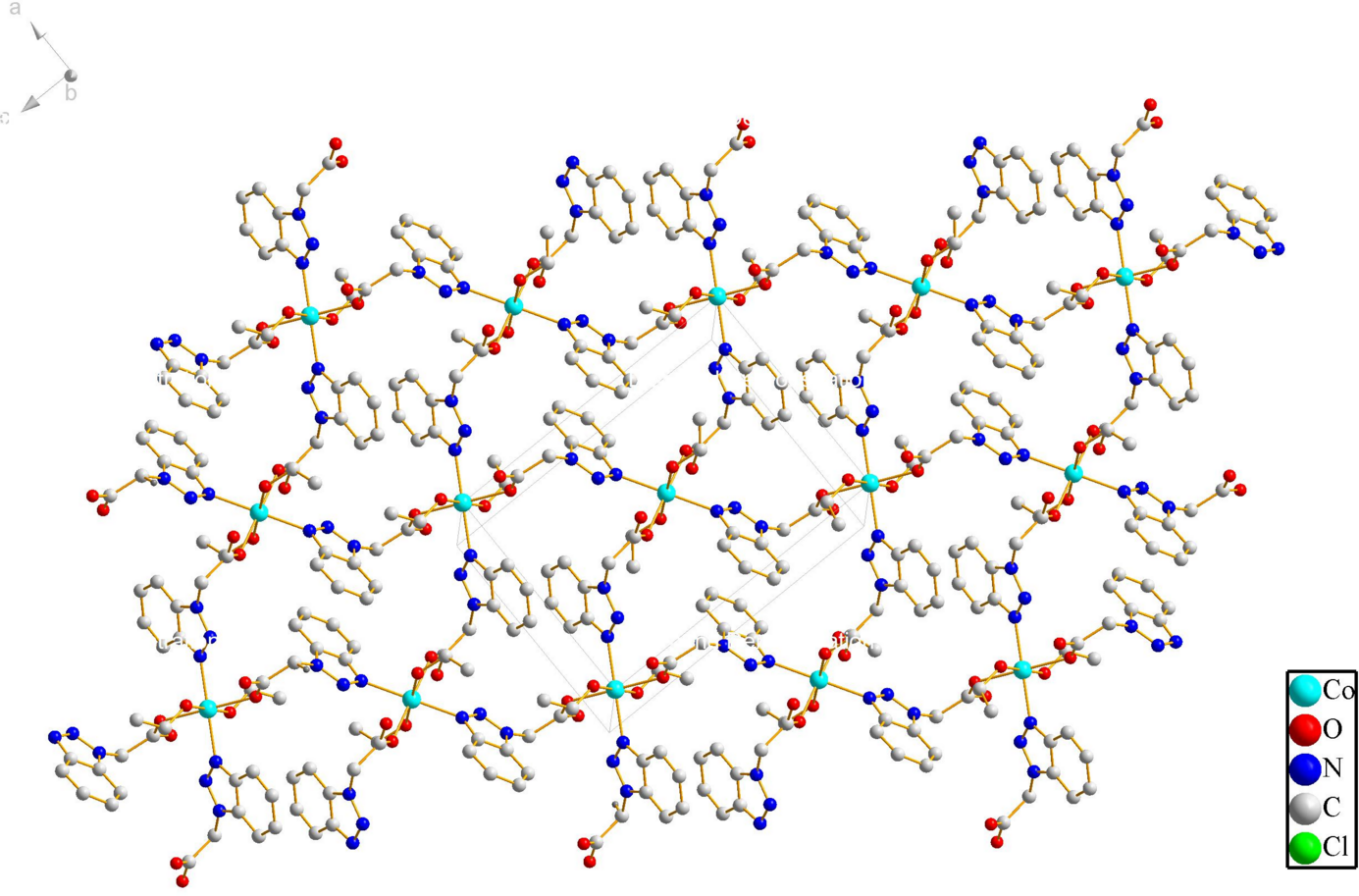
Part of a (010) sheet in the title compound.

**Figure 3:**
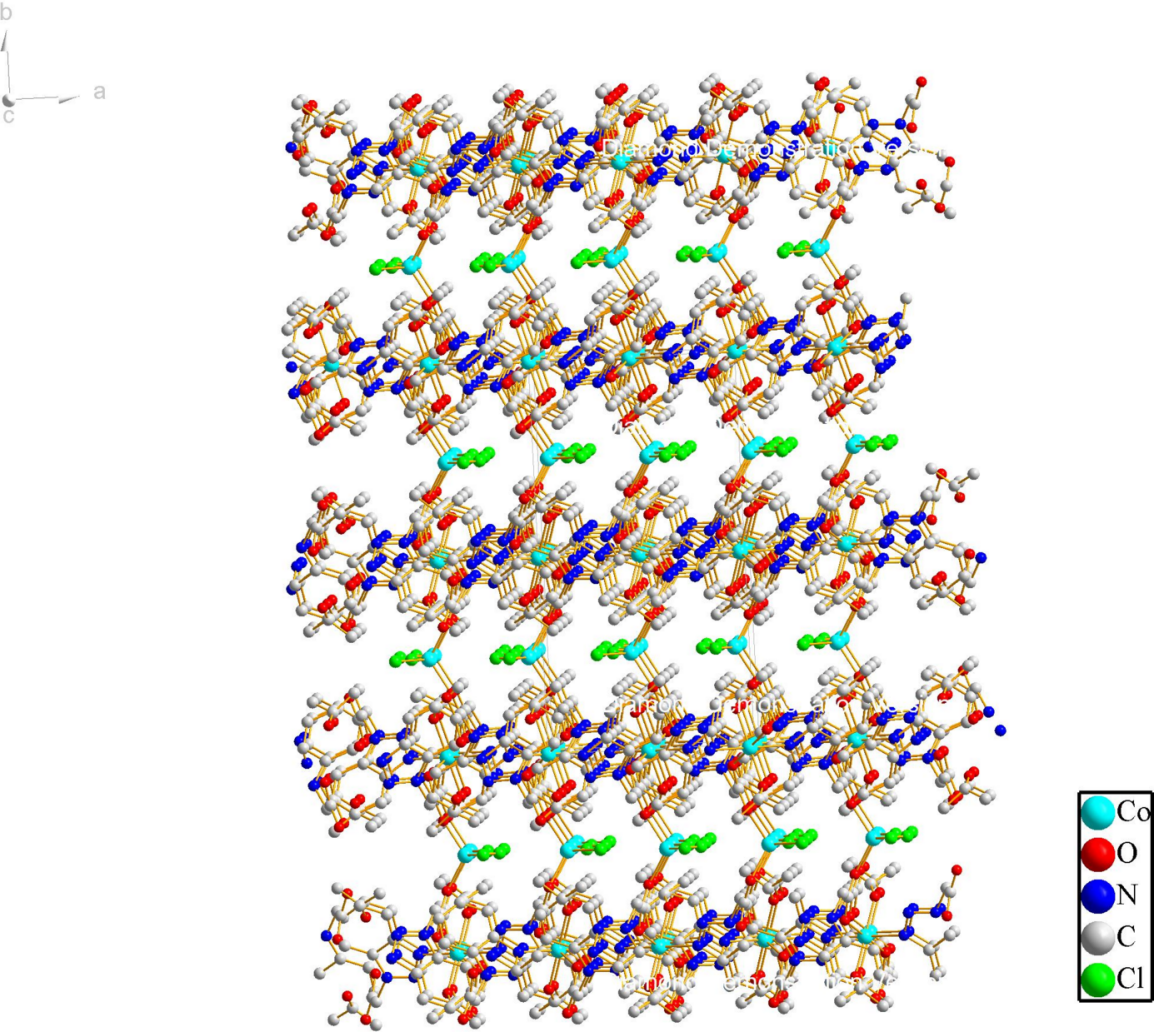
The three-dimensional network in the title compound.

Single-crystal structural analysis reveals that the asymmetric unit consists of two Co^II^ cations (one with site symmetry *m* and one with site symmetry 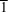), one *L*^−^ ligand, two chloride ions (both site symmetry *m*) and one disordered ethanol mol­ecule (Fig. 1 ▸). Co1 is four-coordinated by two *L*^−^ ligands in O-monodentate mode and two μ^1^-chloride ions in a tetra­hedral coordination geometry, whereas Co2 is six-coordinated by four *L*^−^ ligands (two in N-monodentate mode and two in O-monodentate mode) and two ethanol mol­ecules. In the extended structure, the μ^3^-*O*,*O*,*N* bridging *L*^−^ ligand links the Co2 nodes into (010) sheets (Fig. 2 ▸) and the Co1Cl_2_ fragments link the sheets into a tri-periodic network (Fig. 3 ▸). An O—H⋯O hydrogen bond (Table 1 ▸) occurs.

## Synthesis and crystallization

CoCl_2_ (1.00 mmol) and 2-(benzotriazol-1-yl) acetic acid (1.00 mmol) were mixed in 10.0 ml of ethanol with stirring for about 30 min at room temperature. Blue block-shaped crystals of the title compound were collected by filtration in 40% yield. Analysis (%) calculated (Found) for C_10_H_12_O_3_N_3_ClCo: C, 37.94 (37.72); H, 3.82 (3.89); N, 13.27 (13.32).

## Refinement

Crystal data, data collection and structure refinement details are summarized in Table 2 ▸.

## Supplementary Material

Crystal structure: contains datablock(s) I. DOI: 10.1107/S2414314624006308/hb4477sup1.cif

Structure factors: contains datablock(s) I. DOI: 10.1107/S2414314624006308/hb4477Isup2.hkl

CCDC reference: 2366143

Additional supporting information:  crystallographic information; 3D view; checkCIF report

## Figures and Tables

**Table 1:** Hydrogen-bond geometry (Å, °)

*D*—H⋯*A*	*D*—H	H⋯*A*	*D*⋯*A*	*D*—H⋯*A*
O3—H3⋯O1	0.86 (1)	2.01 (2)	2.734 (3)	141 (2)

**Table 2:** Experimental details

Crystal data	
Chemical formula	[Co(C_8_H_6_N_3_O_2_)Cl(C_2_H_5_OH)]
*M*_r_	316.61
Crystal system, space group	Orthorhombic, *Pnma*
Temperature (K)	223
*a*, *b*, *c* (Å)	9.681 (2), 18.411 (4), 13.163 (3)
*V* (Å^3^)	2346.1 (9)
*Z*	8
Radiation type	Mo *K*α
μ (mm^−1^)	1.69
Crystal size (mm)	0.25 × 0.15 × 0.09

Data collection	
Diffractometer	Bruker SMART CCD
Absorption correction	Multi-scan (*SADABS*; Krause *et al.*, 2015 ▸)
*T*_min_, *T*_max_	0.745, 0.859
No. of measured, independent and observed [*I* > 2σ(*I*)] reflections	13580, 3058, 2337
*R*_int_	0.037
(sin θ/λ)_max_ (Å^−1^)	0.672

Refinement	
*R*[*F*^2^ > 2σ(*F*^2^)], *wR*(*F*^2^), *S*	0.045, 0.120, 1.09
No. of reflections	3058
No. of parameters	184
No. of restraints	3
H-atom treatment	H atoms treated by a mixture of independent and constrained refinement
Δρ_max_, Δρ_min_ (e Å^−3^)	0.71, −0.38

Computer programs: *SMART* and *SAINT* (Bruker, 2002 ▸), *SHELXD1997* and *SHELXL* (Sheldrick, 2008 ▸) and *OLEX2* (Dolomanov *et al.*, 2009 ▸).

## full crystallographic data

### Poly[[µ3-2-(benzotriazol-1-yl)acetato-κ3O:O':N3]chlorido(ethanol-κO)cobalt(II)] Crystal data

**Table:**

[Co(C_8_H_6_N_3_O_2_)Cl(C_2_H_6_O)]	*D*_x_ = 1.793 Mg m^−^^3^
*M**_r_* = 316.61	Mo *K*α radiation, λ = 0.71073 Å
Orthorhombic, *P**n**m**a*	Cell parameters from 39979 reflections
*a* = 9.681 (2) Å	θ = 2.6–27.6°
*b* = 18.411 (4) Å	µ = 1.69 mm^−^^1^
*c* = 13.163 (3) Å	*T* = 223 K
*V* = 2346.1 (9) Å^3^	Block, blue
*Z* = 8	0.25 × 0.15 × 0.09 mm
*F*(000) = 1288	

### Poly[[µ3-2-(benzotriazol-1-yl)acetato-κ3O:O':N3]chlorido(ethanol-κO)cobalt(II)] Data collection

**Table:**

Bruker SMART CCD diffractometer	2337 reflections with *I* > 2σ(*I*)
ω scans	*R*_int_ = 0.037
Absorption correction: multi-scan (SADABS; Krause *et al.*, 2015)	θ_max_ = 28.5°, θ_min_ = 1.9°
*T*_min_ = 0.745, *T*_max_ = 0.859	*h* = −12→13
13580 measured reflections	*k* = −23→24
3058 independent reflections	*l* = −11→17

### Poly[[µ3-2-(benzotriazol-1-yl)acetato-κ3O:O':N3]chlorido(ethanol-κO)cobalt(II)] Refinement

**Table:**

Refinement on *F*^2^	Primary atom site location: dual
Least-squares matrix: full	Hydrogen site location: mixed
*R*[*F*^2^ > 2σ(*F*^2^)] = 0.045	H atoms treated by a mixture of independent and constrained refinement
*wR*(*F*^2^) = 0.120	*w* = 1/[σ^2^(*F*_o_^2^) + (0.0582*P*)^2^ + 2.3224*P*] where *P* = (*F*_o_^2^ + 2*F*_c_^2^)/3
*S* = 1.09	(Δ/σ)_max_ < 0.001
3058 reflections	Δρ_max_ = 0.71 e Å^−^^3^
184 parameters	Δρ_min_ = −0.38 e Å^−^^3^
3 restraints	

### Poly[[µ3-2-(benzotriazol-1-yl)acetato-κ3O:O':N3]chlorido(ethanol-κO)cobalt(II)] Special details

**Table:**

Geometry. All esds (except the esd in the dihedral angle between two l.s. planes) are estimated using the full covariance matrix. The cell esds are taken into account individually in the estimation of esds in distances, angles and torsion angles; correlations between esds in cell parameters are only used when they are defined by crystal symmetry. An approximate (isotropic) treatment of cell esds is used for estimating esds involving l.s. planes.

### Poly[[µ3-2-(benzotriazol-1-yl)acetato-κ3O:O':N3]chlorido(ethanol-κO)cobalt(II)] Fractional atomic coordinates and isotropic or equivalent isotropic displacement parameters (Å^2^)

**Table:**

	*x*	*y*	*z*	*U*_iso_*/*U*_eq_	Occ. (<1)
Co1	0.43288 (6)	0.2500	0.34271 (4)	0.03019 (17)	
Co2	0.5000	0.5000	0.5000	0.02529 (16)	
Cl1	0.34331 (13)	0.2500	0.18726 (8)	0.0404 (3)	
Cl2	0.27129 (14)	0.2500	0.46492 (10)	0.0472 (3)	
O1	0.5318 (2)	0.33968 (11)	0.37700 (15)	0.0296 (4)	
O2	0.5907 (2)	0.45529 (11)	0.36795 (15)	0.0288 (4)	
O3	0.4442 (3)	0.40048 (12)	0.55516 (16)	0.0404 (6)	
H3	0.448 (4)	0.3656 (8)	0.5120 (12)	0.061*	
N1	0.6491 (2)	0.44636 (13)	0.16425 (17)	0.0279 (5)	
N2	0.7812 (3)	0.44389 (13)	0.13833 (18)	0.0303 (5)	
N3	0.8091 (3)	0.50073 (13)	0.08376 (18)	0.0296 (5)	
C1	0.5701 (3)	0.39542 (15)	0.3296 (2)	0.0245 (6)	
C2	0.4659 (6)	0.3692 (2)	0.6509 (3)	0.0657 (13)	
H2AA	0.4721	0.4093	0.6986	0.079*	0.507 (11)
H2AB	0.3817	0.3432	0.6674	0.079*	0.507 (11)
H2BC	0.5343	0.3314	0.6415	0.079*	0.493 (11)
H2BD	0.5090	0.4063	0.6925	0.079*	0.493 (11)
C3	0.5741 (13)	0.3227 (7)	0.6741 (10)	0.095 (4)	0.507 (11)
H3A	0.6599	0.3487	0.6695	0.143*	0.507 (11)
H3B	0.5747	0.2829	0.6270	0.143*	0.507 (11)
H3C	0.5626	0.3045	0.7419	0.143*	0.507 (11)
C4	0.5894 (3)	0.38538 (16)	0.2171 (2)	0.0303 (6)	
H4A	0.6480	0.3433	0.2062	0.036*	
H4B	0.5002	0.3748	0.1870	0.036*	
C5	0.6926 (3)	0.54205 (16)	0.0755 (2)	0.0297 (6)	
C6	0.5883 (3)	0.50689 (16)	0.1263 (2)	0.0303 (6)	
C7	0.4538 (3)	0.5326 (2)	0.1284 (3)	0.0388 (7)	
H7	0.3835	0.5079	0.1619	0.047*	
C8	0.4311 (4)	0.5955 (2)	0.0789 (3)	0.0464 (9)	
H8	0.3422	0.6146	0.0776	0.056*	
C9	0.5380 (4)	0.6334 (2)	0.0290 (3)	0.0446 (8)	
H9	0.5181	0.6775	−0.0022	0.053*	
C10	0.6678 (4)	0.60771 (17)	0.0254 (2)	0.0389 (7)	
H10	0.7376	0.6324	−0.0087	0.047*	
C3A	0.3657 (10)	0.3405 (5)	0.7065 (6)	0.061 (3)	0.493 (11)
H3AA	0.3169	0.3050	0.6670	0.092*	0.493 (11)
H3AB	0.3030	0.3781	0.7272	0.092*	0.493 (11)
H3AC	0.4047	0.3178	0.7655	0.092*	0.493 (11)

### Poly[[µ3-2-(benzotriazol-1-yl)acetato-κ3O:O':N3]chlorido(ethanol-κO)cobalt(II)] Atomic displacement parameters (Å^2^)

**Table:**

	*U*^11^	*U*^22^	*U*^33^	*U*^12^	*U*^13^	*U*^23^
Co1	0.0432 (4)	0.0220 (3)	0.0254 (3)	0.000	0.0007 (2)	0.000
Co2	0.0336 (3)	0.0231 (3)	0.0191 (3)	−0.0040 (2)	−0.0011 (2)	−0.0030 (2)
Cl1	0.0500 (7)	0.0401 (6)	0.0310 (6)	0.000	−0.0062 (5)	0.000
Cl2	0.0544 (7)	0.0450 (7)	0.0423 (6)	0.000	0.0154 (6)	0.000
O1	0.0409 (11)	0.0250 (10)	0.0230 (10)	−0.0056 (9)	0.0008 (9)	0.0008 (8)
O2	0.0389 (11)	0.0259 (10)	0.0217 (9)	−0.0043 (8)	0.0033 (8)	−0.0050 (8)
O3	0.0704 (16)	0.0263 (11)	0.0245 (11)	−0.0106 (11)	−0.0037 (11)	−0.0014 (9)
N1	0.0327 (12)	0.0291 (12)	0.0220 (11)	−0.0024 (10)	0.0040 (10)	0.0015 (9)
N2	0.0369 (13)	0.0287 (12)	0.0252 (12)	0.0001 (11)	0.0060 (10)	0.0035 (10)
N3	0.0351 (13)	0.0273 (12)	0.0265 (12)	−0.0005 (10)	0.0046 (11)	0.0059 (10)
C1	0.0249 (13)	0.0263 (14)	0.0223 (13)	0.0001 (11)	−0.0018 (11)	0.0015 (10)
C2	0.112 (4)	0.047 (2)	0.038 (2)	−0.001 (2)	−0.014 (2)	0.0075 (18)
C3	0.111 (10)	0.079 (8)	0.096 (9)	0.013 (7)	−0.004 (7)	0.027 (6)
C4	0.0430 (16)	0.0242 (13)	0.0238 (13)	−0.0065 (12)	0.0059 (12)	−0.0004 (11)
C5	0.0386 (16)	0.0295 (14)	0.0210 (13)	−0.0006 (12)	0.0021 (12)	0.0013 (11)
C6	0.0385 (15)	0.0307 (15)	0.0217 (13)	0.0012 (12)	0.0007 (12)	−0.0015 (11)
C7	0.0369 (16)	0.0463 (19)	0.0332 (17)	0.0021 (15)	0.0027 (14)	−0.0039 (15)
C8	0.047 (2)	0.054 (2)	0.0388 (19)	0.0146 (17)	−0.0046 (16)	−0.0047 (16)
C9	0.062 (2)	0.0367 (18)	0.0351 (18)	0.0117 (16)	−0.0053 (16)	0.0043 (15)
C10	0.055 (2)	0.0334 (16)	0.0281 (15)	0.0023 (15)	0.0004 (15)	0.0046 (13)
C3A	0.082 (6)	0.069 (6)	0.033 (4)	−0.019 (5)	0.003 (4)	0.007 (4)

### Poly[[µ3-2-(benzotriazol-1-yl)acetato-κ3O:O':N3]chlorido(ethanol-κO)cobalt(II)] Geometric parameters (Å, º)

**Table:**

Co1—Cl1	2.2223 (13)	C2—H2BC	0.9700
Co1—Cl2	2.2439 (14)	C2—H2BD	0.9700
Co1—O1^i^	1.961 (2)	C2—C3	1.387 (12)
Co1—O1	1.961 (2)	C2—C3A	1.324 (9)
Co2—O2	2.114 (2)	C3—H3A	0.9600
Co2—O2^ii^	2.114 (2)	C3—H3B	0.9600
Co2—O3^ii^	2.043 (2)	C3—H3C	0.9600
Co2—O3	2.043 (2)	C4—H4A	0.9700
Co2—N3^iii^	2.152 (3)	C4—H4B	0.9700
Co2—N3^iv^	2.152 (3)	C5—C6	1.373 (4)
O1—C1	1.257 (3)	C5—C10	1.397 (4)
O2—C1	1.228 (3)	C6—C7	1.386 (5)
O3—H3	0.859 (9)	C7—H7	0.9300
O3—C2	1.401 (4)	C7—C8	1.347 (5)
N1—N2	1.324 (3)	C8—H8	0.9300
N1—C4	1.441 (4)	C8—C9	1.410 (5)
N1—C6	1.356 (4)	C9—H9	0.9300
N2—N3	1.298 (3)	C9—C10	1.344 (5)
N3—Co2^v^	2.152 (2)	C10—H10	0.9300
N3—C5	1.365 (4)	C3A—H3AA	0.9600
C1—C4	1.505 (4)	C3A—H3AB	0.9600
C2—H2AA	0.9700	C3A—H3AC	0.9600
C2—H2AB	0.9700		
			
Cl1—Co1—Cl2	112.83 (6)	C3—C2—O3	124.4 (7)
O1—Co1—Cl1	113.73 (6)	C3—C2—H2AA	106.2
O1^i^—Co1—Cl1	113.73 (6)	C3—C2—H2AB	106.2
O1—Co1—Cl2	100.10 (7)	C3A—C2—O3	123.4 (6)
O1^i^—Co1—Cl2	100.10 (7)	C3A—C2—H2BC	106.5
O1—Co1—O1^i^	114.66 (13)	C3A—C2—H2BD	106.5
O2^ii^—Co2—O2	180.0	C2—C3—H3A	109.5
O2^ii^—Co2—N3^iii^	93.56 (9)	C2—C3—H3B	109.5
O2—Co2—N3^iii^	86.44 (9)	C2—C3—H3C	109.5
O2^ii^—Co2—N3^iv^	86.44 (9)	H3A—C3—H3B	109.5
O2—Co2—N3^iv^	93.56 (9)	H3A—C3—H3C	109.5
O3—Co2—O2	93.03 (8)	H3B—C3—H3C	109.5
O3^ii^—Co2—O2^ii^	93.03 (8)	N1—C4—C1	115.4 (2)
O3^ii^—Co2—O2	86.97 (8)	N1—C4—H4A	108.4
O3—Co2—O2^ii^	86.97 (8)	N1—C4—H4B	108.4
O3^ii^—Co2—O3	180.0	C1—C4—H4A	108.4
O3—Co2—N3^iii^	87.74 (10)	C1—C4—H4B	108.4
O3—Co2—N3^iv^	92.26 (10)	H4A—C4—H4B	107.5
O3^ii^—Co2—N3^iii^	92.25 (10)	N3—C5—C6	107.8 (2)
O3^ii^—Co2—N3^iv^	87.75 (10)	N3—C5—C10	131.4 (3)
N3^iii^—Co2—N3^iv^	180.0	C6—C5—C10	120.8 (3)
C1—O1—Co1	135.83 (19)	N1—C6—C5	104.4 (3)
C1—O2—Co2	128.27 (18)	N1—C6—C7	133.0 (3)
Co2—O3—H3	115.0 (14)	C5—C6—C7	122.6 (3)
C2—O3—Co2	130.4 (2)	C6—C7—H7	122.0
C2—O3—H3	106.3 (14)	C8—C7—C6	115.9 (3)
N2—N1—C4	119.0 (2)	C8—C7—H7	122.0
N2—N1—C6	110.6 (2)	C7—C8—H8	119.0
C6—N1—C4	130.1 (3)	C7—C8—C9	122.1 (3)
N3—N2—N1	108.4 (2)	C9—C8—H8	119.0
N2—N3—Co2^v^	117.20 (19)	C8—C9—H9	119.0
N2—N3—C5	108.8 (2)	C10—C9—C8	121.9 (3)
C5—N3—Co2^v^	132.23 (19)	C10—C9—H9	119.0
O1—C1—C4	115.1 (2)	C5—C10—H10	121.7
O2—C1—O1	125.2 (3)	C9—C10—C5	116.6 (3)
O2—C1—C4	119.6 (2)	C9—C10—H10	121.7
O3—C2—H2AA	106.2	C2—C3A—H3AA	109.5
O3—C2—H2AB	106.2	C2—C3A—H3AB	109.5
O3—C2—H2BC	106.5	C2—C3A—H3AC	109.5
O3—C2—H2BD	106.5	H3AA—C3A—H3AB	109.5
H2AA—C2—H2AB	106.4	H3AA—C3A—H3AC	109.5
H2BC—C2—H2BD	106.5	H3AB—C3A—H3AC	109.5
			
Co1—O1—C1—O2	154.9 (2)	N2—N3—C5—C10	−179.9 (3)
Co1—O1—C1—C4	−24.5 (4)	N3—C5—C6—N1	1.1 (3)
Co2—O2—C1—O1	−27.3 (4)	N3—C5—C6—C7	−176.7 (3)
Co2—O2—C1—C4	152.1 (2)	N3—C5—C10—C9	177.7 (3)
Co2—O3—C2—C3	−96.0 (8)	C4—N1—N2—N3	174.0 (2)
Co2—O3—C2—C3A	127.9 (6)	C4—N1—C6—C5	−174.1 (3)
Co2^v^—N3—C5—C6	162.5 (2)	C4—N1—C6—C7	3.3 (5)
Co2^v^—N3—C5—C10	−16.0 (5)	C5—C6—C7—C8	−1.3 (5)
O1—C1—C4—N1	−172.8 (3)	C6—N1—N2—N3	−0.5 (3)
O2—C1—C4—N1	7.8 (4)	C6—N1—C4—C1	−84.3 (4)
N1—N2—N3—Co2^v^	−165.54 (18)	C6—C5—C10—C9	−0.7 (5)
N1—N2—N3—C5	1.1 (3)	C6—C7—C8—C9	−0.8 (5)
N1—C6—C7—C8	−178.3 (3)	C7—C8—C9—C10	2.1 (6)
N2—N1—C4—C1	102.4 (3)	C8—C9—C10—C5	−1.3 (5)
N2—N1—C6—C5	−0.4 (3)	C10—C5—C6—N1	179.8 (3)
N2—N1—C6—C7	177.0 (3)	C10—C5—C6—C7	2.0 (5)
N2—N3—C5—C6	−1.4 (3)		

Symmetry codes: (i) *x*, −*y*+1/2, *z*; (ii) −*x*+1, −*y*+1, −*z*+1; (iii) *x*−1/2, *y*, −*z*+1/2; (iv) −*x*+3/2, −*y*+1, *z*+1/2; (v) −*x*+3/2, −*y*+1, *z*−1/2.

### Poly[[µ3-2-(benzotriazol-1-yl)acetato-κ3O:O':N3]chlorido(ethanol-κO)cobalt(II)] Hydrogen-bond geometry (Å, º)

**Table:**

*D*—H···*A*	*D*—H	H···*A*	*D*···*A*	*D*—H···*A*
O3—H3···O1	0.86 (1)	2.01 (2)	2.734 (3)	141 (2)
